# A bio-psycho-social exercise program (RÜCKGEWINN) for chronic low back pain in rehabilitation aftercare - Study protocol for a randomised controlled trial

**DOI:** 10.1186/1471-2474-11-266

**Published:** 2010-11-17

**Authors:** Christian Hentschke, Jana Hofmann, Klaus Pfeifer

**Affiliations:** 1 Institut of Sport Science, Friedrich-Alexander-University Erlangen-Nuremberg, Gebbertstraße 123b, 91058 Erlangen, Germany

## Abstract

**Background:**

There is strong, internationally confirmed evidence for the short-term effectiveness of multimodal interdisciplinary specific treatment programs for chronic back pain. However, the verification of long-term sustainability of achieved effects is missing so far. For long-term improvement of pain and functional ability high intervention intensity or high volume seems to be necessary (> 100 therapy hours). Especially in chronic back pain rehabilitation, purposefully refined aftercare treatments offer the possibility to intensify positive effects or to increase their sustainability. However, quality assured goal-conscious specific aftercare programs for the rehabilitation of chronic back pain are absent.

**Methods/Design:**

This study aims to examine the efficacy of a specially developed bio-psycho-social chronic back pain specific aftercare intervention (RÜCKGEWINN) in comparison to the current usual aftercare (IRENA) and a control group that is given an educational booklet addressing pain-conditioned functional ability and back pain episodes. Overall rehabilitation effects as well as predictors for compliance to the aftercare programs are analysed. Therefore, a multicenter prospective 3-armed randomised controlled trial is conducted. 456 participants will be consecutively enrolled in inpatient and outpatient rehabilitation and assigned to either one of the three study arms. Outcomes are measured before and after rehabilitation. Aftercare programs are assessed at ten month follow up after dismissal form rehabilitation.

**Discussion:**

Special methodological and logistic challenges are to be mastered in this trial, which accrue from the interconnection of aftercare interventions to their residential district and the fact that the proportion of patients who take part in aftercare programs is low. The usability of the aftercare program is based on the transference into the routine care and is also reinforced by developed manuals with structured contents, media and material for organisation assistance as well as training manuals for therapists in the aftercare.

**Trial Registration:**

Trial Registration number: NCT01070849

## Background

Chronic back pain (cbp) is one of the most frequent reasons for rehabilitation assignment in Germany and associated with high socio-economical burdens [1,2]. The main purpose of rehabilitation is the reduction of individual impairment of functions, activities and participation in social life [3-6]. Consistent with the ICF as underlying classification system in a bio-psycho-social understanding, the recreation of functional health as a key factor stands in the foreground of the efforts [3]. In this sense, pain-conditioned functional ability and back pain episodes are important proximal goals, which in turn should improve also important, more distal outcomes like the restoration of workability and the enhancement of self-determination. In order to be able to plan the achievement of intended results systematically, regarding desired outcomes, rehabilitation must meet quality criteria concerning structures, processes and outcomes [7,8]. Therefore, the choice of sub-goals, contents and methods should be based on the scientific proof of their effectiveness.

Referring to at least one of the outcomes, pain-conditioned functional ability, back pain episodes, health-related quality of life and days of sick leave, some evidence is reported for the short-term effectiveness of inpatient multimodal and interdisciplinary rehabilitation programs [9-15]. These rehabilitative interventions try to consider relevant factors of the chronification process of back pain [16-19]. Hence they target:

• the influence on subjective theories on back pain,

• the reduction of fear-avoidance behaviour,

• the modification of pain coping strategies and further psychological risk factors as well as

• the compensation of physical deconditioning and the enhancement of physical activity and

• the improvement of muscular stabilisation of the spine.

Based upon the underlying evidence, these objectives are also considered to be important in current treatment recommendations [20-22]. For mediation of these sub-goals, exercise therapy takes an important part in the rehabilitation process of chronic back pain, holding a great proportion of overall rehabilitation time in Germany [23-25].

However, the verification of long-term sustainability of achieved effects is missing so far [13]. For a long-term improvement of pain and functional ability, high intervention intensity or high volume, respectively, seems to be necessary (> 100 therapy hours), although it is not known in which period of time this volume has to be provided [13,20,24]. For the same reason of non-satisfying long-term evidence, general effectiveness of inpatient rehabilitation for chronic back pain in Germany has been put into question [11,26]. In available national mainly uncontrolled studies, merly short-term rehabilitation effects with relatively low effect sizes are reported [11]. To date, only Dibbelt et al. were able to report more constant higher effect sizes for a multimodal rehabilitation program [15]. But these effects also were cut back in comparison to control group. As reasons for missing long-term effects of inpatient rehabilitation in Germany, different causes have been discussed by Hüppe et al., concerning masked effects, non-satisfying multimodal profile of treatment, missing individualization of treatment, insufficient treatment intensity and inadequate aftercare [11]. Taking into account the high intensity or volume that seems to be necessary for the improvement of relevant outcomes in chronic back pain, the last two mentioned reasons deserve particular attention. Apparently, inpatient and outpatient rehabilitation intensity or duration is not sufficient to accomplish enduring effects on desirable outcomes in chronic back pain. Nevertheless, considering necessary adaptations in the recommended relevant target areas and specific sub-goals for effective rehabilitation programs, this is not really surprising. Within the typical scope of mostly three to four weeks of rehabilitative intervention in Germany, long-lasting adaptations referring to physical capabilities as well as health-related behaviour patterns may be initiated, but are hardly achieved to the full extent [27-29]. However, in the sense of recreation of functional health, exactly these adaptations in physical capabilities and in health-related behaviour patterns are assumed to form the basis of positive health benefits [6,30]. Gerdes et al. go even further with their statement for rehabilitation aftercare in Germany [31]. They postulate that the real rehabilitation process just begins after the institutional phase is completed.

Purposefully refined aftercare programs offer the possibility to intensify positive effects of inpatient and outpatient rehabilitation or to increase their sustainability [31-33]. In order to be capable of answering these expectations, aftercare programs should aim at the intended outcomes systematically [7,8]. Therefore, implicit or explicit assumptions about the intended impact, processes must involve the relevant determinants of desired change in appropriate intervention programs [8]. Until further notice, for chronic back pain these determinants concern foremost the same objectives, target areas, contents and methods that have proven to be relevant or effective, respectively, in the institutional phase of rehabilitation as postulated in current recommendations and described above. Although in order to contrive durable effects, the relevance of strategies to encourage adherence to health-related behaviour increases. For aftercare interventions the specificity concerning the mentioned determinants inherently supposes an alignment at the necessities of chronic back pain as medical indication. Nevertheless, no indication-related, quality assured, specific aftercare program currently exists for persons with chronic back pain in Germany. This indicates the need for the implementation and evaluation of a suitable, indication-related, quality assured aftercare program.

For the purpose of implementing an aftercare program for chronic back pain, exercise therapy offers a broad approach, because of its inherent multidimensional structure. As a main therapy module in general rehabilitation as well as in rehabilitation of chronic back pain, it could provide improvement of pain-conditioned functional ability and back pain episodes by mediating adaptations in the relevant target areas. Furthermore, an exercise therapy based aftercare program, completed with parts of motivational and volitional aspects, has the potential to produce enduring health enhancing effects by persistently increasing health related physical activity [28,29,34].

On the basis of this assumption, an aftercare intervention relying on an existing modular concept was developed [35,36]. This aftercare program (in German: Rückengesundheit - Wirksamkeit bewegungs- und verhaltensbezogener Interventionen in der Nachsorge, RÜCKGEWINN) obeys existing quality criterions and current recommendations for interventions with chronic back pain based on scientific evidence and should therefore improve the individual success of treatment [20-22,37].

## Objectives

The main purpose of the present study is to examine the efficacy of the developed bio-psycho-social aftercare intervention program for chronic back pain (RÜCKGEWINN) in comparison to current usual aftercare (IRENA) and a control group that is given an educational booklet addressing pain-conditioned functional ability and back pain episodes. Secondary objectives concern the program induced changes of other factors relevant for active self management, for example pain-related cognition like catastrophizing, or physical activity, and their influence on the mentioned primary outcomes as well as their efficacy controlled for empirically proved risk factors (yellow flags). An additional objective is the illumination of mediated operant mechanisms of the aftercare programs.

## Methods/Design

### Study Design

**Figure 1:**
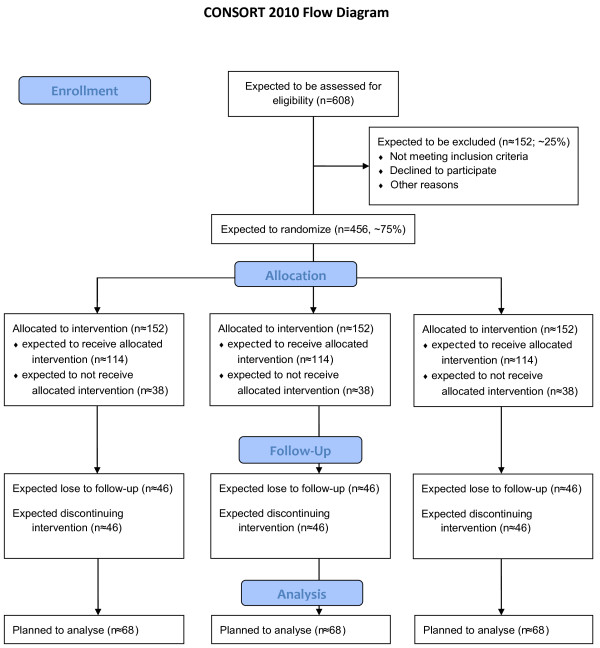
**Study design and planned/expected number of cases according to CONSORT Statement**.

The study is designed as multicenter prospective randomised controlled trial in a three-factorial, split-plot plan (3x3xn) characterized as profile analysis (Figure 1) [38]. Thereby "aftercare treatment" appears as whole-plot factor with three categories respectively study arms (booklet, IRENA, RÜCKGEWINN) crossed with the sub-plot time factor that has also three categories (before and after rehabilitation and 12 month follow-up) [39]. The aftercare treatments will be carried out between 2^nd ^and 3^rd ^measurement. The third factor is the nested patient factor and is treated as random [40]. In addition, we stratify and control the trial for the covariables "chronicity staging", "gender", "rehabilitation facility" and "aftercare facility". Therefore, these covariables appear as additional factors.

### Outcomes

Primary outcome is pain-conditioned functional ability as a direct expression of disability and core outcome component in treatments for chronic pain, measured with the Hannover Functional Ability Questionnaire (FFbH-R) [41-43]. The FFbH-R consists of 12 items with a three-stage answering scale. Its summary score describes the back pain-related functional ability in activities of daily living (ADLs) in adults on a scale of 0% (minimum functional ability) to 100% (maximum functional ability). The questionnaire is constructed for response to already light and moderate functional restrictions. The average item-intercorrelation amounts to 0.50. The test-retest-reliability with repeated measures after approximately one week is above 0.75. Cronbach alpha figures 0.90 [43]. The one factorial structure of the instrument could be confirmed in a principal component analysis. The comparison with related constructs and instruments (Health Assessment Questionnaire, Roland Morris Questionnaire, MOPO scales, Pain Disability index) resulted in steady correlations of 0.75 and greater.

Secondary outcome is the average pain intensity during the last six month measured with a NRS (numeric rating scale) ranging from 0 to 10 [42,44,45]. As sensitivity analysis the von Korff pain grading system is used, which also includes constructs of pain-conditioned functional ability and pain intensity [46].

Further, secondary outcomes refer to factors relevant to active self management and address cognitive, emotional and behavioural coping strategies, physical activity and aftercare adherence behaviour. The latter includes motivational and volitional factors, depression, quality of life assessment, social demographic variables and days of sick leave. All outcomes and the way they are measured are shown in table 1[43-62].

**Table 1:** primary and secondary study outcomes and assessment instruments

outcome/construct	measuring instrument	Literature
**physical disability**		
pain-conditioned functional ability	Hannover Functional Ability Questionnaire	[43]
**pain measures**		
number of pain days	Graded Cronic Pain Status (GCPS)	
pain history functional (dis-)ability (1 Item)		[46]
pain intensity	Numeric Rating Scale (NRS)	[45]
**coping strategies, psychic disability and fear-avoidance beliefs**		
pain coping strategies	Questionaire for detection of	[48]
pain-conditioned psychic disability	pain coping strategies (FESV)	
pain-related fear-avoidance and endurance coping strategies	Avoidence-Endurance Questionaire (AEQ)	[49]
fear-avoidance beliefs	Tempa Scale of Kinesiophobia	[50,51]
catastrophizing	Pain Catastrophing Scale (PCS)	[52,53]
**physical activity, motivational and volitional factors**		
physical activity	Freiburg Questionaire of physical activity	[54]
intention, self-efficacy, HAPA-stage	Health Process Action Approach (HAPA)	[29,55]
**depression and quality of life assessment**		
quality of life assessment	SF-12	[56,57]
depression	PHQ-D	[58]
generalized anxiety disorder	GAD-7	[59]
work satisfaction	IRES-3	[60]
**basic and social demographical variables**		
workability in days of sick leave in past 6 month	Graded Cronic Pain Status (GCPS)	[46]
work load at current employment hospitalization frequency	social demographical rehabilitation core data-set	[61]
other social demographical variables		
chronicity staging	Mainz Pain Staging System (MPSS)	[62]
Aftercare participation	self developed questionaire items & attendance list	**-**

Additionally, for each lesson, the participants' attendance and their perceived disability on a one item NRS is assessed in the two active study arms [47].

### Participants

The population we intend to examine are persons with a history of recurrent or enduring back pain episodes due to an unclear or unspecific cause which does not sufficiently explain the extent of experienced pain. Therefore 456 participants will be consecutively enrolled from inpatient and outpatient chronic back pain rehabilitation in six cooperating rehabilitation facilities, which cover the rehabilitative care of Berlin. For inclusion criteria the ICD-10 is used. We included the following diagnoses, in which back pain of unclear or unspecific cause is frequently encoded in medical practice: M51.2 - M51.4, M51.8 - M51.9 (other disk herniation), M53.8 - M53.9 (other specified/unspecified dorsopathies), M54.5, M54.8 - M54.9 (low back pain, other dorsalgia), M54.4 (if radicular symptoms are not dominating). Patients with appropriate diagnosis are asked to take part in the study by the responsible physician. Before baseline assessment, an informed consent is taken to obtain the patients' approval and exclusion criteria are certified. For obvious reasons, we formulated the following exclusion criteria, although patients met the mentioned ICD-10 diagnoses:

• specific reason for back pain, based on a clear cause or diagnosis, which could sufficiently explain its extent (e.g. radicular symptomatic, myelopathesis, inflammatory changes in the spinal column etc.)

• already carried out surgery on the spine within the last year

• additional serious psychic diagnosis

• uncorrected serious visual and acoustic disability

• seriously reduced health status (other diseases) with considerable reduction of dexterity

• application for retirement

• low German language skills (to fulfill the questionnaires)

• age less than 18 or over 65

• residential area outside of Berlin

Eligible patients are randomised and allocated to either one of the three aftercare treatments. To support the participant's recruitment process, a flow chart and a guiding paper was generated and provided to the clinic practitioners and staff.

### Sample Size and Power Calculation

The sample size calculation was approximated with a 3x3-factorial ANOVA-approach based on the primary outcome and was done with the software "gpower 3.1" [63,64]. To prove an intervention effect with about medium effect size of Cohen's f = 0.27 with an error probability α = 0.05 and power β = 0.8, n ≈ 68 people in each study arm are required for analysis [65]. This was calculated in view of the chosen factorial design with m = 8 estimated fixed parameters. With a supposed drop-out rate of 40% within the progress of the interventions, we need 114 participants in every study arm to begin the assigned aftercare intervention. In addition, we assume that 75% of all participants that were recruited from inpatient or outpatient rehabilitation will start out well with their assigned aftercare treatment. So we need to recruit 152 participants in every study arm or 456 for total sample size (see also Figure 1).

### Randomisation and Data management

In consideration of the logistic situation with several external and internal recruiting rehabilitation facilities and the provision of aftercare in diverse residential areas with miscellaneous aftercare patients in each aftercare facility, specific demands arise for the data management and the randomisation procedure. To accomplish a realisation with high scientific quality, we chose a largely electronic and internet-supported solution for data management including an online-randomisation feature. We implemented a data base for partly electronic data capturing where participants and patients not participating with the right inclusion diagnosis must be registered by the rehabilitation practitioners via a web-application. For an estimation of the participation ratio, all patients who meet one of the inclusion diagnoses are counted anonymously, independent of their participation in the study or any exclusion criterion. Registration of participants must be done before allocation is accomplished by the study software, so allocation concealment is assured automatically. For sequence generation we used an urn randomisation algorithm [66]. This algorithm is the most widely studied member of the family of adaptive biased-coin designs and provides a good compromise between controlling multivariate experimental bias and balancing the trial [67]. Advantages are good statistical properties, which force a small sized trial to be balanced and approaching complete randomisation as the sample size increases, with less vulnerability to allocation bias than permuted-block design. Consequently, an additional practical capability arises. The urn randomisation allows stratification for either large or small number of covariables with unknown prevalence. In this trial we stratify the randomization for "chronicity staging" with 3 subcategories, "gender" with two subcategories and "rehabilitation facility" with six participating centres.

Further purpose of the electronic data management system is that, as a planning tool and tool for data transfer and communication with corresponding facilities, it allows to support the organisation of the aftercare groups.

### Aftercare Treatments

In the accomplishment of the medical rehabilitation, there will be no deviation from the usual routine treatment, except the described recruitment process. For long term sustainability and the enhancement of the intensity of the rehabilitation process, the investigational aftercare intervention "RÜCKGEWINN" shows formal and didactic divergences from the standard program "IRENA". Due to the fact that also "IRENA" has not been evaluated yet, we included another comparison treatment that has been shown efficacious already [68-70]. All aftercare treatments are shortly described below:

#### a) educational booklet

All participants in this study arm will receive an educational booklet from their rehabilitation practitioner in their dismissal examination as well as the advice to return to normal activities as soon as possible. As educational booklet, the German version of the "back book" of Burton et al. was chosen [68]. This booklet provides information about the new approach to back pain, causes of back pain, dealing with an attack of back pain, risk factors for development of chronic back pain and the role of activity. All information that is provided is in accordance with up-to-date scientific knowledge and is based on a bio-psycho-social model of back pain like described in Waddell [16].

#### b) IRENA

**Figure 2:**
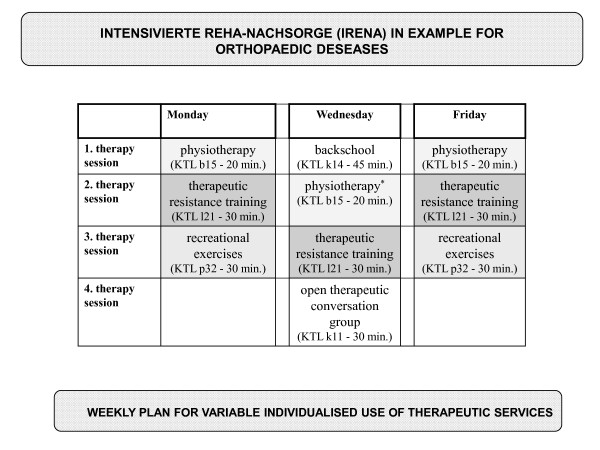
**IRENA weekly therapy plan**.

All participants in this study arm will be introduced into the normal IRENA program (in German: Intensivierte Rehabilitationsnachsorge), which is usual care in Germany [71]. Every patient will be assigned to a certified outpatient aftercare facility near their residential area. Aftercare practitioners and patients can compile an individual therapeutic package from certain appointed therapeutic services [71]. Predominantly resistance training, gymnastics, aquatic exercise, back school and recreation exercises are prescribed by the physicians for aftercare. Most therapies are carried out in open access groups of at least 6 patients without being specific for medical indication. In the IRENA program it is possible to pass the intended 24 exercise sessions with varying frequency per week. Usually, participants complete two or three exercise sessions per week with duration of 90 to 120 minutes per session. Every aftercare facility offers specific therapy combinations at different days a week. Figure 2 shows a weekly therapy plan offered by one of our cooperating facilities.

#### c) RÜCKGEWINN

The RÜCKGEWINN aftercare intervention (in German: Rückengesundheit - Wirksamkeit bewegungs- und verhaltensbezogener Interventionen in der Nachsorge) is the investigational treatment. For its development, we referred to an existing concept and adapted it to aftercare requirements [36]. In order to achieve the intended objectives of improvement and differentiation of the rehabilitation aftercare for chronic back pain patients, three important target areas for multidimensional intervention programs could be deduced from the actual scientific discussion [22,36]. Table 2 shows these target areas and corresponding underlying goals of the RÜCKGEWINN program. To address the above mentioned goals and aspects relevant to chronification, each 90 minutes exercise session interlocks 3 different parts in the mediation process:

**Table 2:** RÜCKGEWINN target areas

target area I: attitude and behavior	
I.a	Modulation of pain- or disease-related subjective theories and the corresponding behavior
I.b	Development of active coping strategies for back pain
I.c	Reduction of psychological pressure with relaxation techniques
I.d	Reduction of fear-avoidance behavior
I.e	Active stabilization of back muscles and reduction of work-load in burdening movements and positions

**target area II: guidance to health enhancing physical activity**	

II.a	Positive change of the attitude to physical activity
II.b	Development of motion skills for independent executed health enhancing physical activity
II.c	Development of control skills for independent executed health enhancing physical activity
II.d	Development of decision-making skills for independent executed health enhancing physical activity
II.e	Development of skills to improve trait and state well being through physical activity and thus reduction of psychological burdens (distress, anxity) and depression

**target area III: improvement of health related physical fitness**	

III.a	Improvement of muscle strength and endurance of back and trunk muscles to prevent states of deconditioning dependent on on inactivity
III.b	Improvement of motor coordination of back and trunk muscles to increase spine stabilization
III.c	Improvement of whole body physical fitness (endurance, flexibility) in sense of enhancement of general physical health resources
III.d	Introduction to several types of physical activity
III.e	Development of a personal network for maintaining physical activity e.g. in fitness facilities

• resource-related mediation of knowledge,

• behavioural modulation and

• physical exercise

We determined the duration of RÜCKGEWINN at 6 months, taking into account the time necessary for the process of behavioural change. In order to have an equal number of sessions compared to IRENA, one session is scheduled weekly for 26 weeks. RÜCKGEWINN will actually be provided in two phases, with the first carried out in 10 consecutive sessions in closed groups. We chose the closed group form, because it is more appropriate to appeal on relevant factors of behavioral change, especially in terms of coping strategies and physical activity enhancement. For anticipated organizational reasons, the second phase of 16 sessions is planned as open access group with partly repeating contents, with the goal set at the participants' empowerment to regular self-determined health enhancing physical activity.

We developed a RÜCKGEWINN manual for therapists and provided some media like for example little ring binders with information cards for participants that will be handed out consecutively each session. We trained several therapists from cooperating certified aftercare facilities to our curriculum. Every patient of this study arm will be allocated to one of those facilities as close as possible to their residence respectively subject to their choice.

### Statistical Hypotheses and Analysis

The primary question refers to the efficacy of the aftercare interventions in comparison to each other. This is related to treatment differences concerning the primary outcome (FFbH-R) during aftercare interventions and quantified by an interaction effect of the corresponding categories of the factors study arm and time between second and third general assessment. Furthermore, the additional single change over time in rehabilitation phases compared to the change effect of aftercare treatment and overall treatment efficacy of rehabilitation plus aftercare intervention should be evaluated for each aftercare treatment. In consideration of adjusting respectively maintaining the multiple α-error for the mentioned aims, multiple hypotheses will be tested in a closed test procedure [72]. Consistent with these objectives and the chosen factorial split-plot design with repeated measures, we use a saturated linear mixed effects model for statistical analysis and stratify for baseline and mentioned covariates [40,73]. To approach the linear change characteristics in two different phases (rehabilitation phases/aftercare treatment) as well as the total change, we use a structured additive linear mixed model with fractional polynomial b-splines with degree l = 1 (linear) and three knots κ_1 _- κ_3_, noted as follows [73]:

I: general form

Y=Uν+Xβ+Zγ+ε

II: reformuled

$$\begin{array}{l} y_{ij}=\beta_{0}+\gamma_{0i}+\beta_{1}t_{ij}+\beta_{2}t_{ij}I_{i}+\beta_{3}t_{ij}R_{i}+\gamma_{1i}t_{ij}+\nu_{1}T+\nu_{2}TI_{i}\\ \;\;+\nu_{3}TR_{i}+\beta_{4}AF_{i}+\beta_{5}RF_{i}+\beta_{6}MPSS_{i}+\beta_{7}G_{i}+\varepsilon_{ij} \end{array}$$

I_i _denotes the indicator variable for IRENA aftercare treatment and R_i _the indicator variable for RÜCKGEWINN aftercare treatment of individual i in a dummy coding with "booklet control" as reference category [73]. The corresponding *β_2 _*and *β_3 _*reflect directly the difference in "slope" of IRENA respectively RÜCKGEWINN to reference "booklet control" and for that reason the efficacy. AF an RF are dummy coded expressions for aftercare respectively rehabilitation facility of patient i, MPSS is also dummy coded variable for chronification stage according to Gerbershagen and G is a dichotomy gender variable. T denotes a suitable construction term for the b-spline for the additional effect (slope) of rehabilitation phases [73].

At first hierarchical, global level in a closed test procedure a likelihood ratio test of the shown specified model against a zero model m_0 _is used with the following global linear hypotheses:

$$H_{0}:C_{AB}\beta =0\;\;H_{1}:C_{AB}\beta \neq 0$$

C is a suitable contrast matrix of fixed interaction effects:

$$C_{AB}=\left(\begin{array}{cccccccc} 0 & 0 & 1 & 0 & 0 & 0 & 0 & 0\\ 0 & 0 & 0 & 1 & 0 & 0 & 0 & 0\\ 0 & 0 & 1 & -1 & 0 & 0 & 0 & 0 \end{array}\right)$$

At second level of the test procedure, three single primary comparisons of change in functional capacity (FFbH-R) during aftercare treatments are performed with *β_i _*~*t(μ,σ^2^)*.:

$$\begin{array}{l} H_{0}:\beta_{2}=0\\ H_{0}:\beta_{3}=0\\ H_{0}:\beta_{2}-\beta_{3}=0 \end{array}\;\;\;\begin{array}{r} H_{1}:\beta_{2}\neq 0\\ H_{1}:\beta_{3}\neq 0\\ H_{1}:\beta_{2}-\beta_{3}\neq 0 \end{array}$$

As shown in the specified model, we consider the stratified analysis to be the primary analysis carried out with the corresponding intention to treat collective. Other analyses are conducted as sensitivity analyses.

To address secondary objectives of the study, the change of secondary outcomes in different aftercare treatments is also assessed. In order to identify effective components or partial goals that should be targeted in aftercare exercise programs, the time varying influence of secondary outcomes on the primary outcomes in the different treatments is modelled by multiple regression modelling.

### Ethical Aspects

The study sponsor being the Deutsche Rentenversicherung Bund (German Federal Pension Fund), which has an official assignment, this study inherently has to accomplish high ethical demands, especially in aspects of data privacy. Additionally, this investigation has been approved by the independent Research Ethics Committee of the Medical Faculty of Friedrich-Alexander-University of Erlangen-Nuremberg on 09.06.2009.

## Discussion

Chronic back pain comes along with a hardly mastered individual disease burden and intrusively affects all areas of life negatively. It has critical consequences on social and occupational participation and causes serious disability. Empirical evidence encourages the relevance of theories for the chronification process of back pain that demand mutually increasing physiological and psychosocial factors that surpass simple additive effect structures (e.g. fear-avoidance model). Thereby, it was shown that particularly by exercise-related and behaviour-related interventions, the appearance as well as the duration of future back pain episodes can be decreased [74-76]. Multidimensional concepts, which combine physical training with cognitive-behavioral components in a bio-psycho-social approach, are appreciated as especially promising [76]. Newer randomized controlled trials confirm these results [77-82]. Thus, we assume that the implementation of a specifically refined aftercare program with a high extent of therapy hours after a stationary rehabilitation leads to a stronger empowerment and a stronger development of self management competence than usual aftercare programs. In detail this should result in reduction of fear-avoidance beliefs as well as maladaptive coping strategies and, on the other side, in enhancement of self-efficacy, a stronger identification with and adherence to physical activity and, as a consequence, a reduction of deconditioning, and a sustainable encouragement of adaptive cognitive and behavioral coping strategies. Hence, we expect substantial and lasting improvements for the majority of the intervention group in view of pain-conditioned functional ability, pain intensity, pain-conditioned psychic disability as well as for subjective general quality of life. The beneficing of a back pain specific aftercare program therefore is obvious, and lies in the transference into the routine care and is also given by developed manuals with structured contents, media and material for organisation assistance as well as training draughts for therapists in aftercare.

Despite the possible high benefit for chronic back pain rehabilitants, special methodological and logistic challenges accrue in this study. A major logistic problem could possibly arise from low rate of patients that begin their prescribed aftercare. In usual aftercare this rate is about 18% concerning inpatient and about 43% concerning outpatient rehabilitation [83]. In this context we have to consider that once patients get back home from rehabilitation, the participation in an aftercare program collides with responsibilities of their daily life. In order to enhance the participation rate, we try to increase the liability of the aftercare for patients with a pre-registration in an aftercare facility when they are still in rehabilitation.

## Competing interests

The authors declare that they have no competing interests.

## Authors' contributions

CH drafted this manuscript and contributed substantially to the final development of the study protocol and study design as well as to the particularizing of the research question relying on the relevant literature. CH was responsible for planning and working out methodically aspects of the study, including e.g. patient enrollment, randomization, assessment and statistical analysis. CH was accountable for planning the implementation of the study, ethical aspects, and data management. CH also contributed to develop the experimental treatment.

JH contributed to develop the research question from national and international literature. JH was responsible for the selection and compilation of the employed questionnaires according to the chosen primary and secondary outcomes. JH contributed to develop the experimental treatment.

KP conceived the study and was responsible for identifying the research question beforehand. KP drafted the study design and was accountable for determining primary and secondary outcomes. KP contrived the basis for development of the experimental treatment.

All authors red and approved the final version of the manuscript.

## Pre-publication history

The pre-publication history for this paper can be accessed here:

http://www.biomedcentral.com/1471-2474/11/266/prepub

## References

[B1] Göbel H Epidemiology and costs of chronic pain syndromes exemplified by specific and unspecific low back pain: Epidemiologie und kosten chronischer schmerzen spezifische und unspezifische Rckenschmerzen Schmerz 2001 15 92 98 10.1007/s004820170031 11810338

[B2] Wenig CM Schmidt CO Kohlmann T Schweikert B Costs of back pain in Germany European Journal of Pain 2009 13 280 286 10.1016/j.ejpain.2008.04.005 18524652

[B3] WHO International Classification of Funtioning, Disability and Health: ICF 2001 Geneva: WHO

[B4] Stucki G Ewert T Cieza A Value and application of the ICF in rehabilitation medicine Disability and Rehabilitation 2003 25 628 634 10.1080/09638280110070221 12959337

[B5] Gerdes N Weis J Bengel J, Koch U Zur Theorie der Rehabilitation Grundlagen der Rehabilitationswissenschaften 2000 Berlin: Springer 41 68

[B6] Pfeifer K Sudeck G Brüggemann S Huber G DGRW-update: Exercise therapy in medical rehabilitation effects, quality, perspectives DGRW-Update: Bewegungstherapie in der medizinischen Rehabilitation Wirkungen, Qualität, Perspektiven 2010 49 224 236 10.1055/s-0030-1261909 20677118

[B7] Donabedian A Evaluating the quality of medical care Milbank Quarterly 2005 83 691 729 10.1111/j.1468-0009.2005.00397.x 16279964 PMC2690293

[B8] Chen H-T Practical Program Evaluation 2005 Thousand Oaks, California: Sage Publications

[B9] Bethge M Müller-Fahrnow W Wirksamkeit einer intensivierten stationären Rehabilitation bei muskuloskelletalen Erkrankungen: systematischer Review und Meta-Analyse Rehabilitation 2008 47 200 209 10.1055/s-2008-1077091 18704869

[B10] Hildebrandt J Pfingsten M Franz C Saur P Seeger D Multidisziplinary treatment programm for chronic low back pain, part 1. Overview. [Das Göttinger Rücken Intensiv Programm (GRIP) - ein multimodales Behandlungsprogramm für Patienten mit chronischen Rückenschmerzen, Teil 1.] Der Schmerz 1996 10 190 203 10.1007/s004820050040 12799853

[B11] Hüppe A Raspe H [Efficacy of inpatient rehabilitation for chronic back pain in Germany: update of a systematic review] Die Rehabilitation 2005 44 24 33 10.1055/s-2004-834602 15668849

[B12] Ostelo RW van Tulder MW Vlaeyen JW Linton SJ Morley SJ Assendelft WJ Behavioural treatment for chronic low-back pain Cochrane Database Syst Rev 2005 1 CD002014 15674889 10.1002/14651858.CD002014.pub2

[B13] van Tulder MW Koes B Malmivaara A Outcome of non-invasive treatment modalities on back pain: an evidence-based review European spine journal: official publication of the European Spine Society, the European Spinal Deformity Society, and the European Section of the Cervical Spine Research Society 2006 15 Suppl 1 S64 81 16320031 10.1007/s00586-005-1048-6 PMC3454555

[B14] Schonstein E Kenny DT Keating J Koes BW Work conditioning, work hardening and functional restoration for workers with back and neck pain Cochrane database of systematic reviews (Online) 2003 12535416 10.1002/14651858.CD001822

[B15] Dibbelt S Greitemann B Büschel C Long-term efficiency of orthopedic rehabilitation in chronic back pain - The integrative orthopedic psychosomatic concept (IopKo) Nachhaltigkeit orthopädischer rehabilitation bei chronischen rückenschmerzen - Das integrierte orthopädisch-psychosomatische behandlungskonzept (IopKo) 2006 45 324 335 10.1055/s-2006-932641 17123214

[B16] Wadell G The back pain revolution 1998 New York: Churchill Livingstone

[B17] Nachemson A Jonsson E Back pain - A scientific enigma in the new millenium Physikalische Medizin Rehabilitationsmedizin Kurortmedizin 2001 11 2 8 10.1055/s-2001-11037

[B18] Nachemson A Jonsson E Neck and back pain: The scientific evidence, causes, diagnosis and treatment 2000 Philadelphia: Lippincott, Williams & Wilkins

[B19] Lühmann D Prävention von Rückenschmerz - Grundlagen und mögliche Interventionsstrategien Bewegungstherapie und Gesundheitssport 2005 21 138 145 10.1055/s-2005-836757

[B20] Guzmán J Esmail R Karjalainen K Malmivaara A Irvin E Bombardier C Multidisciplinary bio-psycho-social rehabilitation for chronic low-back pain Cochrane database of systematic reviews (Online) 2002 CD000963 11869581 10.1002/14651858.CD000963

[B21] Airaksinen O Brox JI Cedraschi C Hildebrandt J Klaber-Moffett J Kovacs F Mannion AF Reis S Staal JB Ursin H Zanoli G Chapter 4: European guidelines for the management of chronic nonspecific low back pain European Spine Journal 2006 15 10.1007/s00586-006-1072-1 16550448 PMC3454542

[B22] Hofmann J Böhle E Bork H Brüggemann S Greitemann B Hildebrandt J Kladny B Pfeifer K Best-practice-recommendations for objectives, contents and methods in the outpatient and inpatient rehabilitation of chronic low back pain Best-Practice-Empfehlungen zu Zielsetzungen, Inhalten und Methoden ambulanter und stationärer Rehabilitationsmaßnahmen von Patienten mit chronifizierenden oder chronischen Rückenschmerzen 1 2010 20 32 39

[B23] Hayden JA van Tulder MW Malmivaara A Koes BW Exercise therapy for treatment of non-specific low back pain Cochrane database of systematic reviews (Online) 2005 CD000335 16034851 10.1002/14651858.CD000335.pub2 PMC10068907

[B24] Hayden JA van Tulder MW Tomlinson G Systematic review: strategies for using exercise therapy to improve outcomes in chronic low back pain Annals of internal medicine 2005 142 776 785 15867410 10.7326/0003-4819-142-9-200505030-00014

[B25] Brüggemann S Sewöster D Tagungsband: DRV-Schriften Bewegungstherapeutische Versorgung in der medizinischen Rehabilitation der Rentenversicherung 19 Rehabilitationswissenschaftliches Kolloquium "Qualität in der Rehabilitation - Management, Praxis, Forschung" vom 8 bis 10 März 2010 in Leipzig 2010 88 378 380

[B26] Raspe H Medical rehabilitation: "change we need": Medizinische Rehabilitation: "Change we need" Die Rehabilitation 2009 48 47 50 10.1055/s-0028-1128122 19206037

[B27] ACSM ACSM's gudelines for exercise testing and prescription 2005 Philadelphia: Lippincott Williams & Wilkins

[B28] Lippke S Ziegelmann JP Health behavior and health behavior change - theories and evidence Applied Psychology 2008 57 541 543 10.1111/j.1464-0597.2008.00338.x

[B29] Schwarzer R Luszczynska A Ziegelmann JP Scholz U Lippke S Social-cognitive predictors of physical exercise adherence: Three longitudinal studies in rehabilitation Health Psychology 2008 27 54 63 10.1037/0278-6133.27.1(Suppl.).S54 18248106

[B30] Weis J Bengel J, Koch U Interventionsmethoden in der Rehabilitation Grundlagen der Rehabilitationswissenschaften 2000 Berlin/Heidelberg/New York: Springer

[B31] Gerdes N Bührlen B Lichtenberg S Jäckel WH Rehabilitationsnachsorge - Analyse der Nachsorgeempfehlungen und ihrer Umsetzung 2005 Regensburg: S. Roderer Verlag

[B32] Deck R Hüppe A Arlt AC Improvement of rehabilitation aftercare through long term follow along of the patients--results of a pilot study: Optimierung der Rehabilitationsnachsorge durch eine längerfristige Begleitung der Rehabilitanden - Ergebnisse einer Pilotstudie Rehabilitation (Stuttg) 2009 48 39 46 19206036 10.1055/s-0028-1105915

[B33] Köpke K-H Upgrade, expand, systematize - An analysis of the status of, the need for reform in, as well as innovative projects for follow-up care in rehabilitation under the German Pension Insurance scheme: Aufwerten, ausbauen und systematisieren - Eine analyse von situation, reformbedarf und innovativen projekten zur nachsorge in der rehabilitation der rentenversicherung Rehabilitation 2005 44 344 352 10.1055/s-2005-915247 16320178

[B34] Jordan JL Holden MA Mason EEJ Foster NE Interventions to improve adherence to exercise for chronic musculoskeletal pain in adults Cochrane Database of Systematic Reviews 2010 1 10.1002/14651858.CD005956.pub2 PMC6769154 20091582

[B35] Pfeifer K Bewegungsbezogene Interventionen zur Förderung der RückengesundheitEntwicklung eines multimodalen Programms im Auftrag der Bertelsmann-Stiftung und der Akademie für Manuelle Medizin 2005 Gütersloh: unpublished

[B36] Pfeifer K Hänsel F Heinz B Rückengesundheit: Grundlagen und Module zur Planung von Kursen 2007 Köln: Dt. Ärzte-Verl

[B37] Pfeifer K Steib S Hentschke C Flothow A Sportwissenschaft KDDR Maual, Neue Rückenschule München: Elsevier in press

[B38] Willett JB Singer JD Martin NC The design and analysis of longitudinal studies of development and psychopathology in context: Statistical models and methodological recommendations Development and Psychopathology 1998 10 395 426 10.1017/S0954579498001667 9635230

[B39] Faller H Reusch A Das experimentelle Design bei der Evaluation von Patientenschulungen Praxis Klinische Verhaltensmedizin und Rehabilitation 2004 17 13 18

[B40] Hedeker D Kaplan D An introduction to growth modeling Quantitative Methodology for the Social Science 2004 Thousand Oaks CA: Sage Publications

[B41] Nigles P Outcome measures in pain therapy Bailliere's Clinical Anesthesiology 1998 12 1 18 10.1016/S0950-3501(98)80003-2

[B42] Dworkin RH Turk DC Wyrwich KW Beaton D Cleeland CS Farrar JT Haythornthwaite JA Jensen MP Kerns RD Ader DN Interpreting the Clinical Importance of Treatment Outcomes in Chronic Pain Clinical Trials: IMMPACT Recommendations Journal of Pain 2008 9 105 121 10.1016/j.jpain.2007.09.005 18055266

[B43] Kohlmann T Raspe H The Hannover Functional Ability Questionnaire for measuring back pain-related functional limitations (FFbH-R) DER FUNKTIONSFRAGEBOGEN HANNOVER ZUR ALLTAGSNAHEN DIAGNOSTIK DER FUNKTIONSBEEINTRACHTIGUNG DURCH RUCKENSCHMERZEN (FFBH-R) 1996 35 I VIII 8693180

[B44] Jensen MP McFarland CA Increasing the reliability and validity of pain intensity measurement on chronic pain paitents Pain 1993 55 195 203 10.1016/0304-3959(93)90148-I 8309709

[B45] Farrar JT Clinical importance of changes in chronic pain intensity measured on an 11-point numerical pain raiting scale Pain 2001 94 149 158 10.1016/S0304-3959(01)00349-9 11690728

[B46] von Korff M Ormel J Keefe FJ Dworkin SF Grading the severity of chronic pain Pain 1992 50 133 149 10.1016/0304-3959(92)90154-4 1408309

[B47] Boonstra AM Schiphorst Preuper HR Reneman MF Posthumus JB Stewart RE Reliability and validity of the visual analogue scale for disability in patients with chronic musculoskeletal pain Int J Rehabil Res 2008 31 165 169 10.1097/MRR.0b013e3282fc0f93 18467932

[B48] Geissner E Fragebogen zur Erfassung der Schmerzverarbeitung FESV Book Fragebogen zur Erfassung der Schmerzverarbeitung FESV 2001 Göttingen: Hogrefe

[B49] Hasenbring M Hallner D Rusu A Fear-avoidance- and endurance-related responses to pain: Development and validation of the Avoidance-Endurance Questionnaire (AEQ) European Journal of Pain 2009 13 620 628 10.1016/j.ejpain.2008.11.001 19101182

[B50] Kori SH Miller RP Todd DD Kinesiophobia: a new view of chronic pain behavior Pain Management 1990 3 35 42

[B51] Vlaeyen JW Kole-Snijders AM Boeren RG van Eek H Fear of movement/(re)injury in chronic low back pain and its relation to behavioral performance Pain 1995 62 363 372 10.1016/0304-3959(94)00279-N 8657437

[B52] Sullivan MJL Bishop S Pivik J The pain catastrophizing scale: development and validation Psychological Assessment 1995 14 432 524

[B53] Van Damme S Crombez G Bijttebier P Goubert L Van Houdenhove B A confirmatory factor analysis of the Pain Catastrophing Scale: invariant factor structure across clinical and non-clinical populations Pain 2002 96 319 324 10.1016/S0304-3959(01)00463-8 11973004

[B54] Frey I Berg A Grathwohl D Keul J Freiburger Fragebogen zur körperlichen Aktivität - Entwicklung, Prüfung und Anwendung Social and Preventive Medicine 1999 44 55 64 10407953 10.1007/BF01667127

[B55] Lippke S Ziegelmann JP Understanding and modeling health behavior change: The multi-stage model of health behavior change Journal of Health Psychology 2006 11 37 50 10.1177/1359105306058845 16314379

[B56] Bullinger M Schuntermann FM Erfassung der gesundheitsbezogenen Lebensqualität mit dem SF-36 Health Survey Rehabilitation 1996 35 XVII XXX 8975342

[B57] Gandek B Ware J Aaronson N Apolone G Bjorner J Brazier J Bullinger M Kaasa s Leplege A Prieto L Sullivan M Cross-validation of item selection and scoring for the SF-12 Health Survey in nine countries: Results from the IQOLA Project Journal of Clinical Epidemiology 1998 51 1171 1178 10.1016/S0895-4356(98)00109-7 9817135

[B58] Löwe B Spitzer RL Zipfel S Herzog W Prime MD Patient Health Questionaire (PHQ-D). Manual 2002 2 Heidelberg/New York: Pfitzer

[B59] Löwe B Decker O Müller S Brähler E Herzog W Schellberg D Herzberg Y Validation and Standardization of the Generalized Anxiety Disorder Screener (GAD-7) in the General Population Medical Care 2008 46 266 274 10.1097/MLR.0b013e318160d093 18388841

[B60] Bührlen B Gerdes N Jäckel WH Entwicklung und psychometrische Testung eines Patientenfragebogens für die medizinische Rehabilitation (IRES-3) Rehabilitation 2005 44 63 74 10.1055/s-2004-834687 15789288

[B61] Deck R Röckelein E Zur Erhebung soziodemographischer und sozialmedizinischer Indikatoren in den rehabilitationswissenschaftlichen Forschungsverbünden Förderschwerpunkt „Rehabilitationswissenschaften" - Empfehlungen der Arbeitsgruppen „Generische Methoden", „Routinedaten" und „Reha-Ökonomie" 1999 Frankfurt/M: Verband Deutscher Rentenversicherungsträger (VDR) 84 102

[B62] Gerbershagen HU Klinger D The Mainz Pain Staging System Antidepressiva als Analgetika 1998 Vienna: Arachne Verlag 71 95

[B63] Faul F Erdfelder E Lang A-G Buchner A G*Power 3: A flexible statistical power analysis program for the social, behavioral, and biomedical sciences Behavior Research Methods 2007 39 175 191 17695343 10.3758/bf03193146

[B64] Faul F Erdfelder E Buchner A Lang A-G Statistical power analyses using G*Power 3.1: Tests for correlation and regression analyses Behavior Research Methods 2009 41 1149 1160 10.3758/BRM.41.4.1149 19897823

[B65] Cohen J Statistical power analysis for the behavioral sciences 1988 2 Hillsdale, NY: Lawrence Erlbaum Associates

[B66] Wei LJ Lachin JM Properties of the urn randomization in clinical trials Controlled Clinical Trials 1988 9 345 364 10.1016/0197-2456(88)90048-7 3203525

[B67] Stout RL Wirtz PW Carbonari JP Del Boca FK Ensuring balanced distribution of prognostic factors in treatment outcome research Journal of Studies on Alcohol 1994 55 70 75 10.15288/jsas.1994.s12.70 7723001

[B68] Burton AK Wadell G Tilloston KM Summerton N Information and advice to patients with back pain can have a positive effect Spine 1999 24 2484 2491 10.1097/00007632-199912010-00010 10626311

[B69] Roberts L Little P Chapman J Cantrell T Pickering R Langridge J The back home trial: general practitioner-supported leaflets may change back pain behavior Spine 2002 27 1821 1828 10.1097/00007632-200209010-00002 12221342

[B70] Kovacs F Abraira V Santos S Diaz E Gestoso M Muriel A Gil Del Real MT Mufraggi N Noguera J Zamora J A comparison of two short education programs for improving low back pain-related disability in the elderly: A cluster randomized controlled trial Spine 2007 32 1053 1059 10.1097/01.brs.0000261556.84266.0f 17471084

[B71] Rahmenkonzeption "Intensivierte Rehabilitations-Nachsorge Deutsche Rentenversicherung Bund 2006

[B72] Marcus R Peritz E Gabriel KR On closed testing procedures with special reference to ordered analysis of variance Biometrika 1976 63 655 660 10.1093/biomet/63.3.655

[B73] Fahrmeir L Kneib T Lang S Regression. Modelle, Methoden und Anwendungen 2007 Berlin, Heidelberg: Spinger-Verlag

[B74] Linton SJ van Tulder MW Preventive interventions for back and neck pain problems? Spine 2001 26 778 787 10.1097/00007632-200104010-00019 11295900

[B75] Kool J de Bie R Oesch P Knüsel O van den Brandt P Bachmann S Exercise reduces sick leave in patients with non-acute non-specific low back pain: a meta-analysis Journal of Rehabilitation Medicine: official Journal of the UEMS European Board of Physical and Rehabilitation Medicine 2004 36 49 62 15180219 10.1080/16501970310020104

[B76] Burton AK Balague F Cardon G Eriksen HR Hanninen O Harvey E Henrotin Y Indahl A Lahad A Leclerc A How to prevent low back pain Best Practise and Research in Clinical Rheumatology 2005 19 541 555 10.1016/j.berh.2005.03.001 15949775

[B77] Jensen IB Bergström G Ljungquist T Bodin L A 3-year follow-up of a multidisciplinary rehabilitation programme for back and neck pain Pain 2005 115 273 283 10.1016/j.pain.2005.03.005 15911154

[B78] Hurwitz EL Morgenstern H Chiao C Effects of recreational physical activity and back exercises on low back pain and psychological distress: Findings from the UCLA low back pain study American Journal of Public Health 2005 95 1817 1824 10.2105/AJPH.2004.052993 16186460 PMC1449442

[B79] Linton SJ Nordin E A 5-year follow-up evaluation of the health and economic consequences of an early cognitive behavioral intervention for back pain: A randomized, controlled trial Spine 2006 31 853 858 10.1097/01.brs.0000209258.42037.02 16622371

[B80] Heymans MW de Vet HCW Bongers PM Knol DL Koes BW van Mechelen W The effectiveness of high-intensity versus loe-intensity back schools in an occupational setting: A pragmatic randomized controlled trial Spine 2006 31 1075 1082 10.1097/01.brs.0000216443.46783.4d 16648740

[B81] Göhner W Schlicht W Preventing chronic back pain: Evaluation of a theory-based cognitive-behavioral training programme for patients with subacute back pain Patient Education and Counseling 2006 64 87 95 10.1016/j.pec.2005.11.018 16540279

[B82] Kool J Bachmann S Oesch P Knüsel O Ambergen T De Bie R van den Brandt P Function-centered rehabilitation increases work days in patients with nonacute nonspecific low back pain: 1-year results from a randomized controlled trial Archives of Physical Medicine and Rehabilitation 2007 88 1089 1094 10.1016/j.apmr.2007.05.022 17826451

[B83] Lindow B Grünbeck P Nachsorge nach medizinischer Rehabilitation - Wer nimmt welche Leistungen in Anspruch? DRV-Schriften 2008 208 209

